# rTMS Suppresses Tobacco Craving via Enhanced Prefronto‐Striato‐Thalamic Connectivity

**DOI:** 10.1111/adb.70167

**Published:** 2026-05-14

**Authors:** Xin Luo, Xuefeng Ma, Shuang Li, Hong'an Chen, Bo Yang, Yanbin Zheng, Guang‐Heng Dong

**Affiliations:** ^1^ Center for Educational Cognitive Neuroscience, Faculty of Education Yunnan Normal University Kunming Yunnan China; ^2^ Center for Cognition and Brain Disorders The Affiliated Hospital of Hangzhou Normal University Hangzhou Zhejiang China

**Keywords:** DLPFC, functional connectivity, prefrontal–striatal pathways, rTMS, tobacco use disorder

## Abstract

Studies have used repetitive transcranial magnetic stimulation (rTMS) to stimulate the left dorsolateral prefrontal cortex (DLPFC) in patients with tobacco use disorder (TUD); consequently, decreased craving for smoking was observed. However, the neural mechanism underlying this process remains unclear. Functional MRI data were collected from 59 valid TUD participants (31 rTMS and 28 shams) when they performed a Go/No‐go task before and after a continuous 5‐day treatment (rTMS on the left DLPFC). Three approaches of data analyses were performed: event‐related data analyses to identify the brain regions that are associated with rTMS treatment; functional connectivity (FC) analyses among the left DLPFC (the stimulate region) and other survived brain regions after group comparison; FC between the left DLPFC and all brain regions to find couplings among the brain regions. rTMS decreased the craving for smoking in patients with TUD. Comparing with the sham group, the rTMS group showed enhanced brain responses in the bilateral ACC, bilateral caudate and left thalamus to the No‐go smoking cues. Further, FC analyses among the survived brain regions showed that rTMS enhanced the FC in DLPFC–caudate and caudate–left thalamus pathways. Notably, enhanced FC between the DLPFC and bilateral basal ganglia thalamus was observed. The current study demonstrated the effectiveness of rTMS in the treatment of TUD, which is associated with enhanced brain responses that are responsible for executive control and reward processing; it enhanced top‐down control by reshaping the prefrontal–striatal pathways.

## Graphical abstract

This study aimed to investigate the therapeutic effects of rTMS on tobacco use disorder (TUD). The findings demonstrated that participants with TUD exhibited reduced cigarette cravings following treatment, accompanied by a reorganization of the prefrontal–striatal pathway and enhanced top‐down cognitive control.

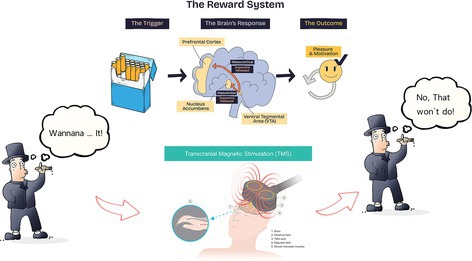

## 1 Introduction

Tobacco use disorder (TUD) remains the leading cause of preventable death globally [1]; however, existing approaches to curbing smoking, including nicotine replacement, medicine and psychotherapy, exhibit a low abstinence rate [2, 3]. Only a few chronic cigarette smokers succeeded in the attempt to quit smoking using the aforementioned approaches [4], even though they are aware of the negative effect of smoking [5]. This evidence suggests that quitting smoking is a challenging process.

Evidence regarding TUD has demonstrated that executive function plays an important role in developing and quitting the habit of smoking [6, 7]. It is assumed that poor executive functions have been linked to nicotine abuse. Further, excessive smoking elicits neuroadaptations, which affect the broadly distributed neural circuits involved in cognitive processes, especially executive control [8]. Researchers have demonstrated that the major cause of relapse is exposure to smoking cues [9], which could elicit the craving for smoking in patients with TUD. Simultaneously, impaired executive control over increased craving for smoking could bring uncontrolled smoking behaviors.

Researchers have used different tasks to explore this issue. For example, Billieux et al. reported the poor ability of patients with TUD to inhibit the craving for smoking in a go–stop task [10]. Moreover, Hu et al. reported that smokers showed lower performance in executive functioning than non‐smokers, which was measured by arithmetic and digit subtests [11]. Flaudias et al. found that impaired inhibition was a strong predictor of TUD [12]. Lerman et al. examined coupling among executive control and default mode networks and found that the inability to disengage from the default mode network may be critical to cognitive or affective alterations that underlie nicotine dependence [13].

Taken together, the key features of patients with TUD described in previous studies suggest that impaired executive functions led to failure in effective control of cravings for smoking, which further led to uncontrolled smoking behaviours in these patients [14]. Hence, it is worthwhile to investigate whether enhancing executive control would help to control cravings for smoking in patients with TUD. Because the highest rates of TUD exist among people with psychiatric disorders, there is a need to consider adapted and innovative treatments for TUD that include non‐invasive brain stimulation. To this end, Muller et al. investigated the effects of repeated frontal tDCS on craving for smoking and executive functions. However, they did not observe improvement in executive functions beyond the placebo effect [15]. Further, it was observed that repetitive transcranial magnetic stimulation (rTMS) on dorsolateral prefrontal cortex (DLPFC) could decrease cravings for smoking; this was highlighted by the attributed role of the lateral prefrontal cortex, which is implicated in controlling the overuse of smoking [16, 17].

Among these innovative treatment strategies, rTMS proved to be an effective strategy. Notably, rTMS administered to the prefrontal cortex has been approved by FDA as a treatment for treatment‐resistant major depression, obsessive‐compulsive disorder and so forth [18, 19, 20]. Studies have demonstrated that rTMS of the prefrontal cortex affects the neural substrate of substance use disorders, including TUD [21, 22, 23]. Previous studies applied focal rTMS over the DLPFC, which is the key region for executive control. This led to a significantly higher quitting rate and reduced cigarette consumption [17, 24, 25] as compared with that in sham. All these results suggest that rTMS could be potentially effective in treating TUD.

Although previous studies have revealed the effectiveness of rTMS on TUD, the neural mechanism underlying this process remains unclear. Particularly, it is worth inquiring why stimulating the DLPFC could generate smoking cessation. Behavioural measures have been used to observe the effect of rTMS on nicotine craving [17, 26], and researchers have proposed that rTMS on the left prefrontal cortex might enhance coupling between executive control and reward processing brain regions [17, 27, 28]. Furthermore, it has been hypothesized that top‐down control over craving underlies the changes parallel to rTMS; however, there is a lack of substantial experimental support regarding this.

The current study aimed to explore the neural changes underlying rTMS in a continuous rTMS course for 5 days. Further, it aimed to find if rTMS could decrease craving for smoking and elucidate the neural changes associated with this process. Specifically, the study aimed to find if rTMS could decrease craving for smoking in patients with TUD, and if this change was associated with enhanced executive control over smoking craving. Furthermore, neural features, including the brain responses to smoking cues and executive control tasks, and the functional connectivity (FC) features among brain regions were investigated.

## 2 Methods and Materials

### 2.1 Ethics

The study conforms to the Code of Ethics of the World Medical Association and was conducted following the principles of the Declaration of Helsinki. The Human Investigations Committee of Hangzhou Normal University approved this research (No. 20190505). All the participants provided written informed consent before the experiment or scan was conducted. The trial protocol has been registered with the Chinese clinical trial registry.

### 2.2 Inclusion and Exclusion Criteria of Patients With TUD

All our studies regarding TUD used the same inclusion and exclusion strategy, which included the following.

First, the inclusion criteria for TUD participants were as follows: (1) smoked at least 10 cigarettes per day, and such smoking lasted 1 year or more; (2) the carbon monoxide (CO) level in the expired air was at least 5 ppm. Expiratory CO levels were measured using the Smokerlyzer System (Bedfont Scientific Ltd., Rochester, UK); (3) scores higher than four in the Fagerström Test for Nicotine Dependence (FTND) that measured the severity of TUD [29, 30]; and (4) scores more than 15 in the 10‐item Tiffney Questionnaire on Smoking Urges (TQSU) [31].

Second, participants had to be motivated to quit (indicated by replies ‘very likely’ or ‘somewhat likely’ to a motivation questionnaire) and must have had no period of abstinence of more than 3 months in the past year. Third, all the participants gave satisfactory answers for a safety screening questionnaire for TMS and fMRI, which included the following: (1) any kind of mental or neurological diseases or related history; (2) the Mini‐International Neuropsychiatric Interview (MINI) showed that participants exhibited no psychiatric disorders [32, 33] suggested that the participants may get depression [34, 35], with cognitive impairment or depression being exclusionary; (3) surgery, head trauma, or heart‐related diseases in the past year; (4) claustrophobia; (5) metal implants and tattoos of the neck or head; (6) any other substance use disorder during the last 12 months before recruitment; (7) use of any psychotropic medication regularly; (8) history of epilepsy or seizures or increased risk of seizures for any reason.

### 2.3 Groups

**FIGURE 1:**
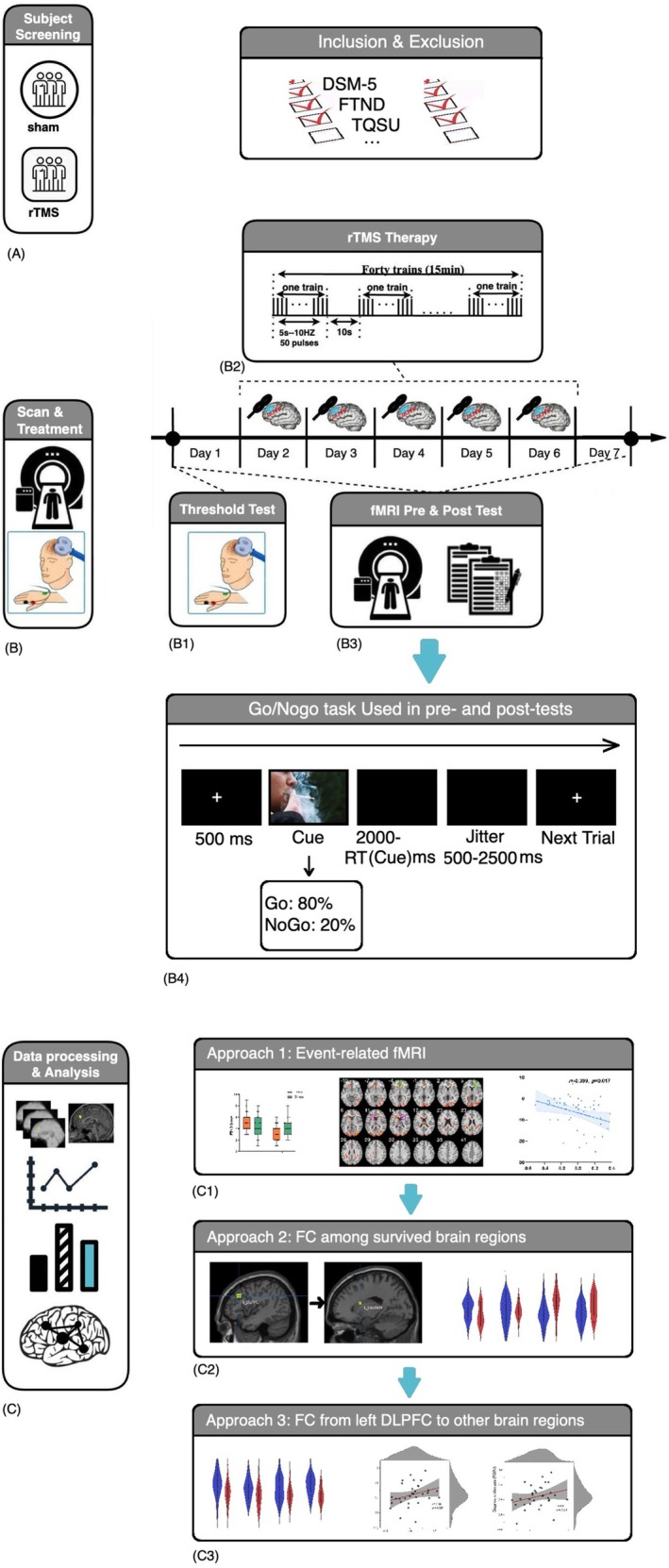
**The framework of the whole research.** (A) Patients were selected based on a series of criteria and were randomized into rTMS and sham groups. (B) The scan and treatment procedure of the current study, including (B1) the safety and threshold test, (B2) continuous rTMS treatment for 5 days, (B3) pre‐ and post‐tests before and after the treatment and (B4) Go/No‐go task used in pre‐ and post‐tests. (C, C1–C3) The approaches for data analyses in the current study.

All the eligible participants (70) were randomly allocated to two groups as follows: rTMS group, and Sham group (fake stimulation) (Figure 1A). This group division was open to rTMS operators since it was impossible to blind them to the group division; however, the participants were blind to the division. During experiment, some of them drop‐off, and the detailed demographic information for these who finished the whole study is shown in Table 1. After the completion of the study, the participants were informed that they might have received real or sham stimulation. They were instructed to estimate their stimulation condition by choosing between 1 (sham) and 5 (real). The participants rated potential adverse events i.e., negative feelings, mental disorder and physical harm to be associated with the stimulation through ‘yes’ or ‘no’ responses. For those sham groups, we provided 5 days of real stimulation for compensation, if they wanted so.

**TABLE 1:** Demographic features of subjects in the current study.

Item	rTMS (*n* = 31)		Sham (*n* = 28)		*t*	*p*
	*M*	*SD*	*M*	*SD*		
Age	23.48	5.58	22.47	2.32	0.713	> 0.05
Smoking years	6.02	5.30	6.27	4.67	0.242	> 0.05
FTND	5.06	1.61	4.39	1.91	1.320	> 0.05
TQSU	25.26	7.96	26.89	9.28	−0.651	> 0.05

Abbreviations: FTND, Fagerström Test for Nicotine Dependence; *M*, mean; rTMS, repetitive transcranial magnetic stimulation; *SD*, standard deviation; TQSU, Tiffney Questionnaire on Smoking Urges.

### 2.4 The rTMS/Sham Treatment

During the first visit of the participants in the pre‐test, all types of measures were collected, which included safety assessments, TQSU, FTND and fMRI measures (Go–No‐go task, T1, resting state) (Figure 1B). In addition, the TQSU and FTND, as measures of tobacco use, can effectively capture individuals' stable levels of nicotine craving. Furthermore, during the fMRI session, to more strongly elicit smoking‐related psychological responses, participants watched a 5‐min smoking‐related video prior to performing the Go/No‐go task. In the following 5 days, daily rTMS (active or sham) was administered to all the participants. After five continuous days of rTMS, the participants were asked to undergo post‐assessment on the following day (post‐test). The assessments were the same as that of the pre‐test (Figure 1B2,B3).

Upon the administration of rTMS, the participants were not instructed to do any further changes to their regular habits, except for not smoking for at least 1 h before each experimental scan. This brief period of abstinence was ensured so that participants had some degree of baseline craving and responsiveness to cigarette cues, without potential confounding by a ceiling effect from prolonged abstinence.

An rTMS research system (Magstim Super Rapid2, Magstim Co., Whitland, UK) was used with a 70‐mm biphasic figure‐8 coil and a special air‐cooling system in a room close to the MRI scanner room. We determined the resting motor threshold (RMT) of each participant at the beginning of each experimental visit before any exposure or ratings. Individual RMT was determined by positioning the coil 45° over the contralateral area of the skull, which corresponded to the contralateral motor cortex. Thereafter, we adjusted the amplitude of the magnetic pulse during each pulse, until we found the lowest intensity that reliably produced thumb or hand movements in at least 5 of the 10 consecutive trials. We put the Cz point of the 10–20 electroencephalography system as the central location, and we leaned approximately 60° to the left and the M1 area was determined about 1 cm ahead. The left DLPFC region was located by moving the coil 5 cm anterior to the M1 region, and we marked the location during a special hat to keep the same location of stimulation for each participant [36, 37].

#### 2.4.1 Active rTMS Procedures

The treatment was standardized at 100% RMT. Each participant received a continuous 5‐day treatment. Further, a session of stimulation consisted of 40 trains of 10 Hz stimulation, each lasting for 5 s with an interval of 10 s. Each treatment session lasted for 15 min, with a total of 2000 pulses (Figure 1B2).

#### 2.4.2 Sham‐rTMS Procedures

Each participant received an RMT measurement similar to that in the real rTMS group. For stimulation, participants of the sham group were stimulated with the rTMS machine vertically at the stimulated site to promise participants felt they were stimulated (Figure 1B). An identical sham procedure has been used in some previous studies, and it has been demonstrated to be indistinguishable from active stimulation [38].

### 2.5 Go/No‐Go Task (Before and After rTMS Treatment)

In the Go/No‐go task, the participants were asked to respond to one type of stimulus but not to respond to another type of stimulus. The majority of the trials (80%) consisted of a go task, and the remaining trials (20%) required no response, based on a predefined rule or context. Thus, the No‐go target appeared before movement initiation, and there was no delay.

The Go/No‐go task consisted of four blocks—in two of these blocks, the No‐go stimuli were smoking pictures, and the other two pictures were neutral. Furthermore, all the go trials were neutral pictures. These four blocks were ABBA/BAAB/BABA/ABAB randomized. The whole task consisted of 400 trials and lasted for approximately 20 min (Figure 1B4).

The numbers of trials for No‐go cues for smoking/neutral are 40 separately. All stimuli pictures were selected from CAPS, a Chinese affective picture system, and the valence and arousal were matched between smoking and neutral pictures. All pictures were shown in full screen on a 21‐inch monitor. Only No‐go trials in different measures and groups were used for further analyses (No‐go_smoking_ vs. No‐go_neutral_). Figure 1B4 shows the task procedures and the timeline for one trial of the task.

First, subjects were asked to fixate their sight on a cross located at the center of the screen for 500 ms. Then, cues were presented, and subjects were instructed to respond or not by pressing buttons ‘1’ (Go) or no response (No‐go). Each cue lasted for 2000 ms at most and would be terminated by pressing a button. If subjects did not respond for 2000 ms, the trial was considered a missed trial. After pressing the button, a black screen was presented for 2000‐RT ms. Finally, a black screen was shown for 500–2500 ms before the next trial. The average of the ITI is 2000 ms.

### 2.6 fMRI Scan Parameters

Structural images were collected using a T1‐weighted three‐dimensional spoiled gradient‐recalled sequence for the whole brain (176 slices, repetition time = 1700 ms, echo time TE = 3.93 ms, slice thickness = 1.0 mm, skip = 0 mm, flip angle = 15, inversion time = 1100 ms, field of view = 240 × 240 mm, in‐plane resolution = 256 × 256). Functional MRI (task) were performed on a 3T scanner (GE, Sigma), with a gradient‐echo EPI T2* weighted‐sensitive pulse sequence in 33 slices (interleaved sequence, 3 mm thickness, TR = 2000 ms, TE = 30 ms, flip angle 90°, field of view 220 × 220 mm^2^, matrix 64 × 64). The participants of the task were asked to finish the task according to the requirements.

### 2.7 Data Analyses Approach 1: Brain Response Changes in Go/No‐Go Task Associated With rTMS Treatment

#### 2.7.1 Data‐Preprocessing

Functional volumes were slice time‐corrected and realigned using the Statistical Parametric Mapping (SPM) 12 package (http://www.fil.ion.ucl.ac.uk/spm). They were co‐registered and normalized to the Montreal Neurological Institute (MNI) template brain and smoothed with a 6‐mm^3^ isotropic Gaussian kernel. No participants were excluded from the analysis because of head motion, and the exclusion criteria were 3 mm in directional movement or 2° in rotational movement. The detailed procedures and parameters are presented in Data S1.

#### 2.7.2 First‐Level Analyses

The general linear model (GLM) analysis for individual participants was performed by NeuroElf v1.1 (http://neuroelf.net). GLM was applied to identify blood‐oxygen‐level–dependent (BOLD) activation according to brain activities. Different types of trials (Go‐pre, No‐go‐pre, Go‐post, No‐go‐post) were separately convolved with a canonical haemodynamic response function for task regression. The duration of each trial was 4000 ms, and GLMs included a constant term per run. Head motion parameters and a high‐pass filter (0.01–0.1 Hz) for 128 s were included as regressions of no interest. The GLM approach was used to identify voxels that were significantly activated for each event during the response stage.

#### 2.7.3 Second‐Level Analyses

First, we identified voxels that showed a main effect in the No‐go trials as compared with the Go trials. Second, we determined voxels that were significantly different concerning BOLD signals between the post and pre‐tests. We identified clusters of contiguous and significantly different voxels at an uncorrected threshold of *p* < 0.001. Finally, these clusters were tested for cluster‐level family‐wise error (FWE) correction at *p* < 0.05. Specially, the AlphaSim estimation indicated that the clusters' extent of 26 adjoining voxels would achieve an FWE threshold of *p* < 0.005 effectively. The smoothing kernel applied in simulating false‐positive (noise) maps using AlphaSim software was 8.4 mm, which was estimated from residual fields of the contrast maps pooled into the one‐sample *t*‐test.

#### 2.7.4 Statistical Analysis

For behavioural and fMRI analyses, ANOVA analyses with groups (rTMS, sham) × tests (pre‐, post‐) were performed in the current study. Post hoc analyses were performed for further testing.

### 2.8 Results for Data Analyses Approach 1: Behavioural Measures

**FIGURE 2:**
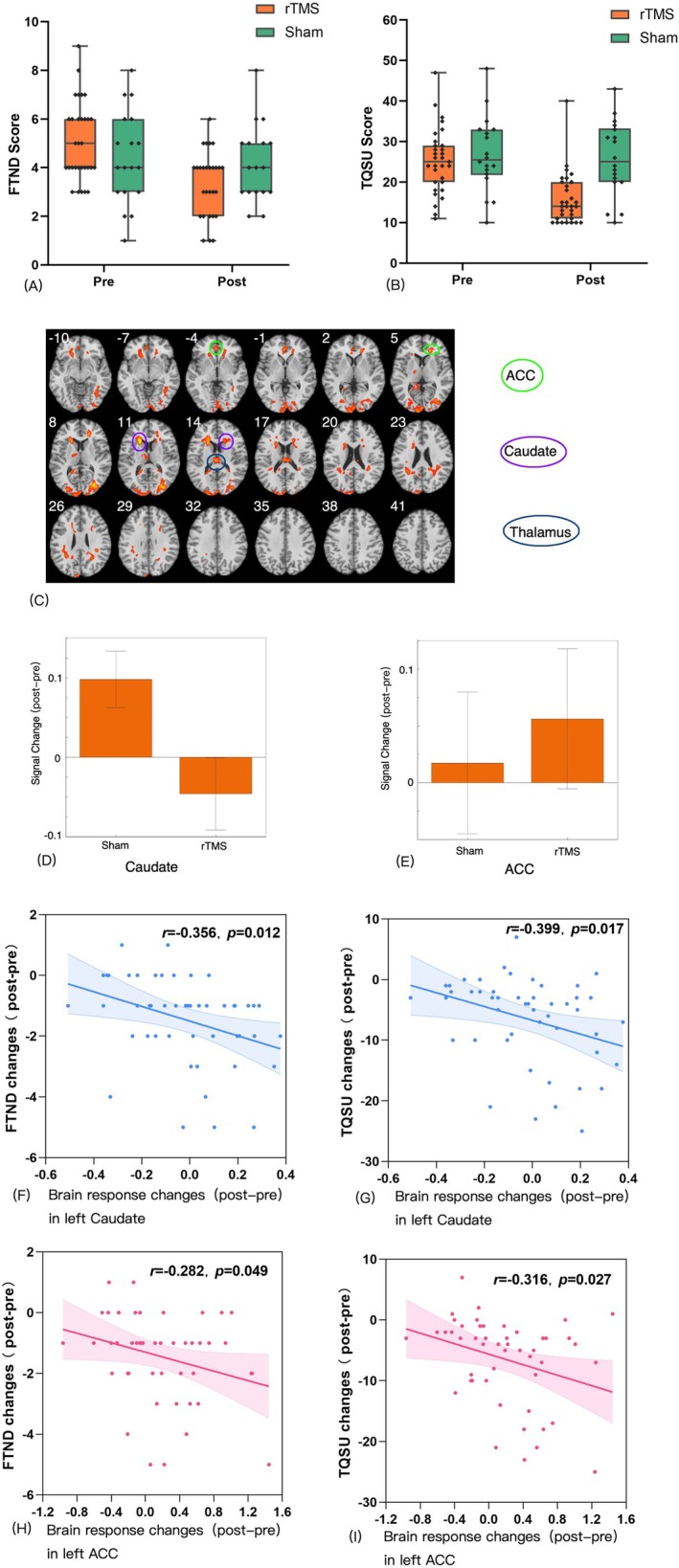
**Behavioural and brain responses to rTMS treatment.** Changes in FTND (A) and TQSU (B) scores before and after rTMS treatment in rTMS and sham groups. (C) Brain responses to No‐go trials in post–pre‐test. (D,E) Beta figures in post–pre comparisons in the caudate and ACC. (F,G) Correlations between brain response changes in post–pre‐test in the left caudate and changes in FTND and TQSU scores. (H,I) Correlations between changes in brain response in post–pre‐test in the left ACC and changes in FTND and TQSU scores.

Regarding FTND, significant group (rTMS, sham) × tests (pre‐, post‐) were observed (*F* = 18.896, *p* < 0.001, *ƞ* ^2^ = 0.287). Further analyses revealed that in the rTMS group, the rTMS decreased the FTND score significantly in post‐tests (pre [*M* = 5.065, *SD* = 0.310] and post [*M* = 3.032, *SD* = 0.305]; *p* < 0.001, *ƞ* ^2^ = 0.221). This effect was not observed in the sham group (pre [*M* = 4.389, *SD* = 0.407] and post [*M* = 3.944, *SD* = 0.401]; *p* = 0.133, *ƞ* ^2^ = 0.104) (Figure 2A). Craving was measured by the TQSU, and similar features were observed in pre‐ and post‐ test scores (Figure 2B) (Find details from Data S1).

#### 2.8.1 Brain Responses

Compared with the sham group, the rTMS group demonstrated enhanced brain responses in the bilateral ACC, bilateral caudate, left thalamus and bilateral occipital gyrus (Figure 2C). No decreased features were observed for brain responses. From the beta figures of these brain regions, we observed that the difference in the caudate region was mostly brought by decreased brain responses in the rTMS group (Figure 2D). However, the differences in ACC were mostly brought by increased brain activation in the rTMS group (Figure 2E). Negative correlations were observed between changes in brain response in the caudate region and FTND (*r* = −0.356, 95% Cl, 0.187 to 0.598, *p* = 0.012) (Figure 2F) and TQSU (*r* = 0.399, 95% Cl, 0.232 to 0.462, *p* = 0.017) (Figure 2G) scores in patients with TUD. Similar features were observed in the left ACC (Figure 2H,I).

### 2.9 Data Analyses Approach 2: FC Changes in the Go/No‐Go Task Associated With rTMS Treatment

To further explain the neural changes associated with rTMS, we analysed FC changes in the Go/No‐go task after rTMS treatment. In this analysis, we used the survived brain regions as ROIs in group comparisons for the Go/No‐go task and calculated their FC features with DLPFC and FCs among the regions.

#### 2.9.1 FC Analyses

Functional volumes were slice time‐corrected and realigned using the Statistical Parametric Mapping (SPM) 12 package (http://www.fil. ion.ucl.ac.uk/spm). Thereafter, images were normalized to the Montreal Neurological Institute (MNI) template brain and spatially smoothed using a full‐width‐at‐half‐maximum Gaussian kernel of 4 mm^3^. No patients were excluded from the analysis because of head motion (the exclusion criteria were 3 mm for directional movement or 2° for rotational movement). We extracted the spherical region‐of‐interest (ROI) with a radius of 4 mm using the WFU_PickAtlas (http://www.ansir.wfubmc.edu) and according to the peak MNI coordinates of the brain regions that survived in Data Analyses 1. Moreover, we calculated further task‐related FC analysis using the CONN toolbox (https:// www.nitrc.org/projects/conn) in MATLAB (onset time equal to the appearance time of the Go/No‐go cues). The CONN toolbox was used to identify principal components associated with segmented cerebrospinal fluid (CSF) and white matter (WM) for each participant. Additional preprocessing steps were identified using the artefact removal toolbox (ART) (https://www. nitrc.org/projects/artifact_detect/), including high‐pass filtering (0.008–0.09 Hz), linear detrending and regression of outlying functional volumes (> 97th percentile in a normative sample; global‐signal *z*‐value threshold = 5, subject‐motion mm threshold = 0.09). Finally, by computing the correlation coefficients between the ROIs, correlation maps of each participant were obtained and converted to Z‐values using Fisher Z transformation. All FC results were corrected using Bonferroni correction.

#### 2.9.2 FC Changes From DLPFC to Brain Regions Showing Group Difference in Go/No‐Go Task

We obtained the survived brain regions as ROIs in group comparisons for the Go/No‐go task and calculated their FC features with DLPFC and the FC among them. In this analysis, ROI selection was based on the group comparison. We used the brain regions that survived in the group comparison, which included the bilateral ACC, bilateral caudate and left thalamus, as ROIs to calculate their FC between different groups.

**FIGURE 3:**
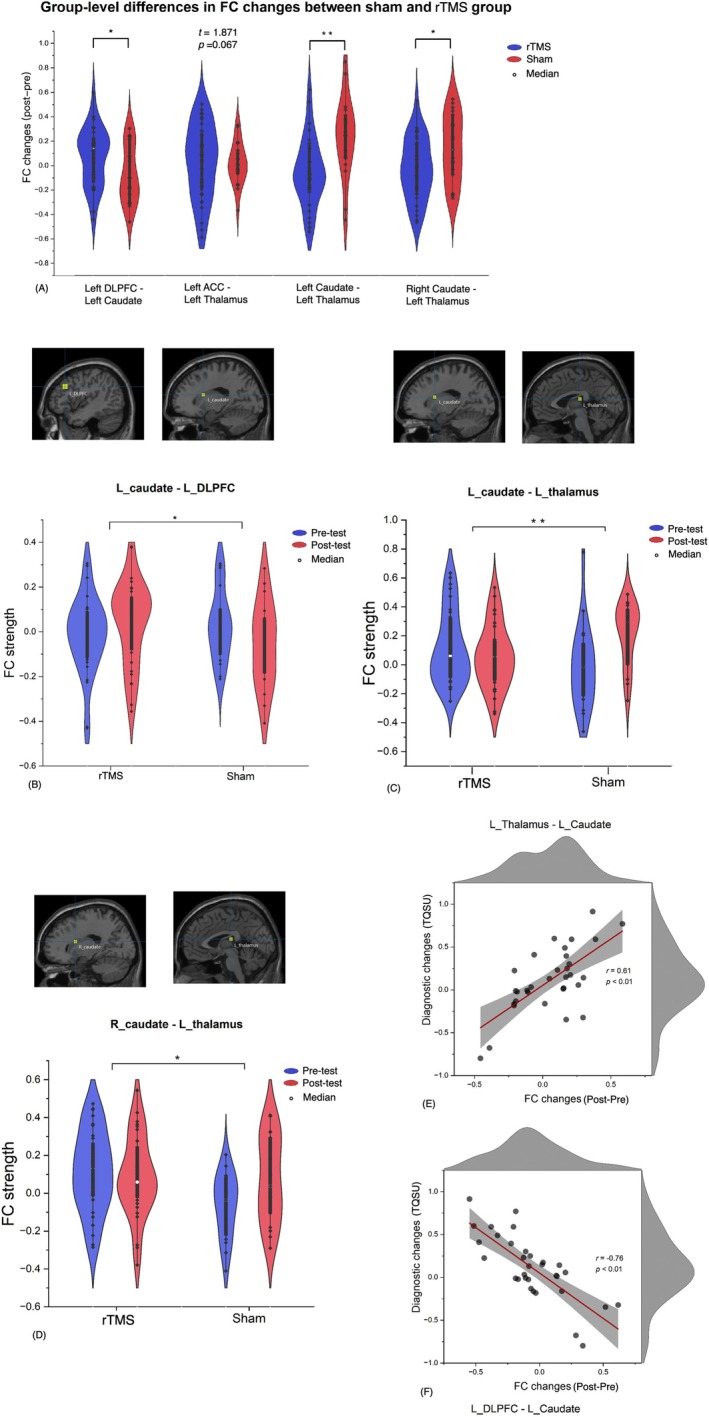
**Functional connectivity changes with rTMS treatment in survived brain regions.** (A) FCs that showed group differences in the post–pre‐test. Detailed FC features between (B) left caudate–left DLPFC, (C) left caudate and left thalamus and (D) right caudate and left thalamus are presented. (E) Correlation between changes in FC in the left thalamus–left caudate and changes in TQSU scores in the rTMS group are presented. (F) Correlation between FC changes in the left DLPFC‐left caudate and changes in TQSU scores in the rTMS group.

We classified this FC into two types: the FC between the executive network and the reward network, which included DLPFC–caudate. The results demonstrated that the changes (post–pre) in the active group were higher than that in the sham group, which indicated that rTMS increased the FC between these regions (Figure 3A,B). Further, it suggested that rTMS treatment increased the coupling between brain regions for executive control and reward. Moreover, FC within the reward network, including left caudate–left thalamus and right caudate–left thalamus, was decreased; further, the FCs among these brain regions also decreased after rTMS treatment, which suggested that rTMS treatment decreased the coupling among brain regions in the reward network (Figure 3A,C,D).

Correlation of these changes in FC values (post–pre) with changes in TQSU scores (post–pre) showed that FC changes between DLPFC–caudate (executive network‐reward network) were negatively correlated with changes in TQSU scores (*r* = −0.76, 95% Cl, −0.327 to −0.822, *p* < 0.01) (Figure 3F), which suggested that more changes (lower score in TQSU changes) were associated with higher FC changes. Furthermore, a positive correlation was also observed between changes in FC between the caudate and thalamus (within the reward network) (*r* = 0.61, 95% Cl, 0.238 to 0.715, *p* < 0.01), which suggested that lower values in TQSU changes (the better) were associated with lower FC among the reward network (the better) (Figure 3E). Correlations with FTND are presented in Table S1.

### 2.10 Data Analysis Approach 3: Neural Changes From Left DLPFC to All Brain Regions

To further explore the neural changes associated with rTMS, we used the left DLPFC (the target of rTMS) as the ROI and calculated the FC changes with all other brain regions associated with rTMS treatment.

#### 2.10.1 FC Changes From DLPFC to the Whole Brain

**FIGURE 4:**
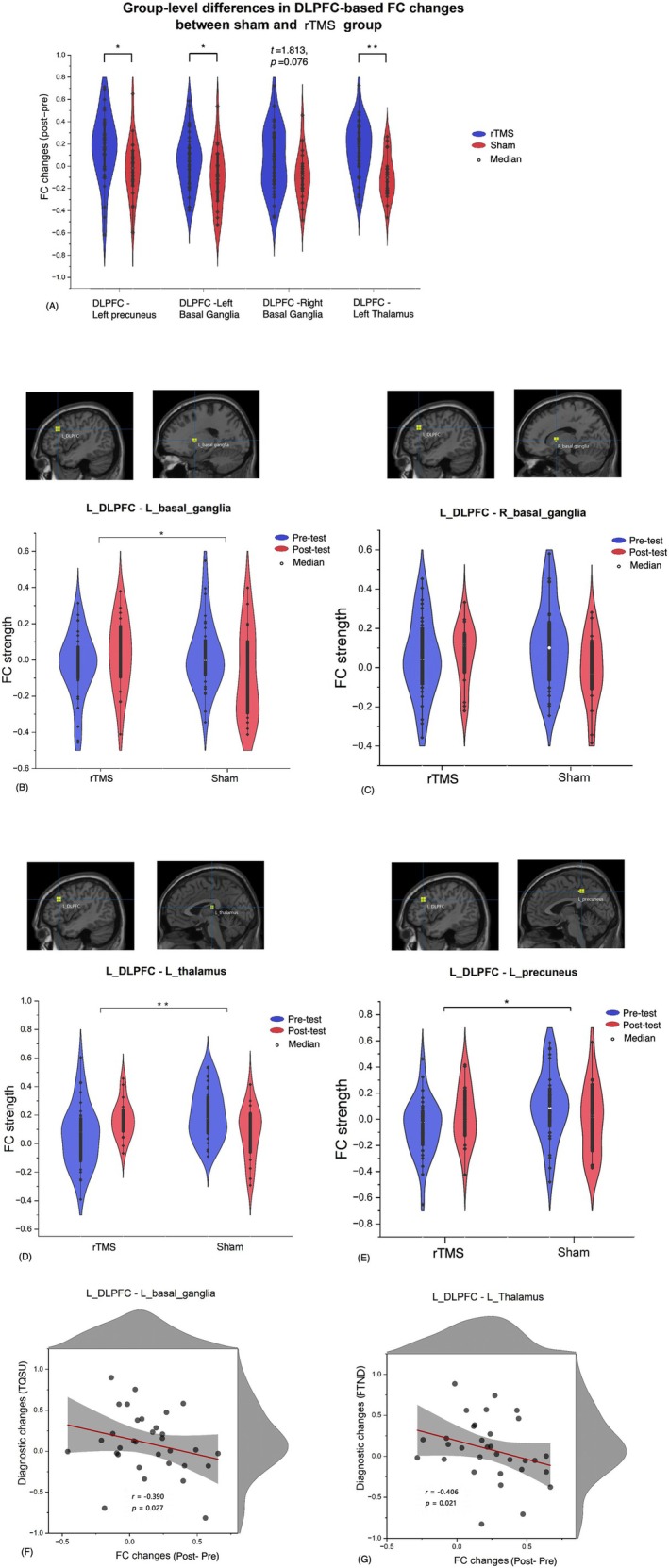
**Functional connectivity changes with rTMS treatment from left DLPFC to other brain regions.** (A) FCs that showed group differences in the post–pre‐test. Detailed FC features between the left DLPFC to the (B) left basal ganglia, to the (C) right basal ganglia, to the (D) left thalamus and to the (E) left precuneus are presented here. (F) Correlation between FC changes in the left DLPFC‐left basal ganglia and changes in TQSU scores in the rTMS group. (G) Correlation between FC changes in the left DLPFC‐left thalamus and changes in TQSU scores in the rTMS group.

The FC analyses were the same as that used in Data Analysis Approach 2 (FC changes in the Go/No‐go task associated with rTMS treatment). The left DLPFC (the target of rTMS) was used as the ROI, and FC changes with the whole brain regions were calculated. We calculated the FC values for post‐test minus pre‐test and compared these values between the rTMS and sham groups. The results demonstrated that rTMS was associated with increased FC between the left DLPFC and bilateral basal ganglia, the thalamus and the left precuneus (Figure 4A). From the features of further comparisons, we can see all the differences were brought by enhanced FC value in the rTMS group (Figure 4B–E).

Correlation analyses between changes in FC (post–pre) and changes in TQSU scores (post–pre) revealed a significant negative association between DLPFC–basal ganglia connectivity (executive network–reward network) and TQSU changes (*r* = −0.390, *p* = 0.027; Figure 4F), indicating that larger increases in FC were associated with more negative changes (i.e., greater reductions) in TQSU scores. In addition, changes in DLPFC–thalamus connectivity were negatively correlated with changes in FTND scores (*r* = −0.406, *p* = 0.021; Figure 4G). Detailed correlation results are provided in Table S2.

## 3 Discussion

Using three approaches for data analyses, the current study investigated the neural changes using rTMS to decrease the craving for smoking in patients with TUD through a Go/No‐go task. We observed that rTMS on the left DLPFC decreased smoking craving, and this process was associated with higher activation in the prefrontal and reward circuit regions while performing the Go/No‐go task. The FC analyses showed that rTMS enhanced the top‐down control by strengthening the frontal‐striatal pathways.

### 3.1 Sham Effects

We observed a strong placebo effect in most of the parameters measured in the current study, which has been well‐documented in previous studies [39]. However, the main effect was observed with ANOVA analyses using group (rTMS, sham) × tests (pre‐, post‐) on the measures—the results showed that rTMS treatment for 5 days consecutively reduced smoking craving significantly as compared with sham treatment.

### 3.2 Continuous 5 Days of rTMS on the Left DLPFC Decreased Craving for Smoking

Behavioural measures showed that rTMS decreased the craving for smoking in patients with TUD, as measured by FTND and TQSU scores. This result further supported that rTMS on the left DLPFC was a useful tool in treating TUD. Further, this corroborates with previous studies on this issue. For instance, Amiaz et al. used high‐frequency rTMS to target the left DLPFC for attenuating nicotine cravings, which were mediated by activation effects [39].

### 3.3 rTMS on the Left DLPFC Enhanced Brain Responses Responsible for Executive Control and Reward in Go/No‐Go Task

Compared with the pre‐test, we observed increased brain activations in the bilateral ACC, bilateral caudate and left thalamus in the Go/No‐go task. The group differences demonstrated that the changes were majorly brought by increased brain responses in the relevant regions for the rTMS group. ACC was proved to be a key region for executive functions, and it is responsible for executive control of behaviours [40, 41]. Moreover, enhanced brain reactivity of these areas to smoking cues suggested that the smokers were trying to inhibit their urge to smoke in the presence of the cues [42]. Studies on rTMS have proved that high‐frequency rTMS (> 5 Hz) could increase brain activity both locally and in distant regions, while low‐frequency TMS (< 1 Hz) can decrease brain activities [43]. In the current study, rTMS on the left DLPFC increased brain reactivity in ACC during gaming No‐go cues, which suggests that rTMS activated the brain regions responsible for executive control functions.

Studies have proved that the caudate region is the key node of the nigrostriatal dopamine circuit that is critical for craving and reward processing [41, 42]. A previous study has shown that high‐frequency rTMS to the human prefrontal cortex could induce the release of dopamine in the caudate nucleus [44]. Further, smoking behaviours and cues can provoke dopaminergic release in the caudate, which is found to be positively associated with self‐reported cravings for smoking [42, 45].

Moreover, rTMS on the left DLPFC also activated the thalamus, which is known to be responsible for craving smoking. It has been proven to play an important role in drug‐seeking [46]. Animal studies have shown that the thalamic nuclei motivate the behaviours in the context of drug‐seeking; moreover, the paraventricular nucleus of the thalamus, the lateral habenula and the mediodorsal nucleus may be involved in the reinstatement, extinction and expression of drug‐seeking behaviours [47, 48]. Human studies have shown that the thalamus is involved in human drug addiction, particularly during drug cue and non‐drug reward processing as well as response inhibition tasks, demonstrating enhanced thalamus activation during a reaction to drug cues and reduced thalamus activation during response inhibition [49, 50, 51].

The negative correlations between changes in brain responses and changes in FTND/TQSU scores suggest that decreased smoking cravings (higher change scores in FTND/TQSU when post–pre) are associated with increased brain activities during reaction to smoking cues. The results in the current study revealed that increased ACC activation was associated with better executive control of smoking cues (lower FTND or TQSU scores), suggesting that high‐frequency rTMS enhanced ACC functions during a reaction to smoking cues. The Go/No‐go task needs participants to control their cravings when exposed to smoking cues, which needs them to engage in more executive control, thereby leading to the activation of the related brain regions.

### 3.4 rTMS Decreased Craving for Smoking by Enhancing Top‐Down Control Over Reward Networks

Notably, rTMS for 5 days also generated effects in regions deep in the brain, that is, the caudate. Because the caudate cannot be directly stimulated with rTMS, our observation suggests that there is a pathway between the focal (left DLPFC) and these regions. To address this, we took the stimulation site (left DLPFC) as the ROI, and performed FC analyses with other survived brain regions and with the whole brain. The results supported the hypothesis that rTMS increased the FC between DLPFC and the caudate, which provided evidence regarding the correlations between executive control and reward processing systems.

The DLPFC is the final projection of the mesolimbic system—it integrates the motivation from the limbic system and cognitive desire from the executive network to inhibit smoking‐seeking behaviours and exerts inhibitory control over other behaviours [40, 52]. When the DLPFC is stimulated with rTMS, the neuro‐adaptations in the reward system that mediates nicotine addiction might get altered, which could be accounted for by the effect of rTMS on cortical excitability [53]. Previous studies have shown that the PFC–striatum pathway plays a key role in cognitive strategies that effectively regulated cravings in smokers [40], which suggests a top‐down control over cue‐induced cravings. The FC results in the current study provided direct evidence regarding this conclusion. Besides this, the changes in brain responses of the caudate were negatively correlated with changes in the FTND/TQSU scores in smokers, suggesting that their enhanced controlling functions were associated with higher brain response in these striatal brain regions, suggesting that the striatum activity fully mediated the relationship between the DLPFC and scores for craving [40]. These results provide direct evidence for the control mechanism of the DLPFC over caudate. The results also suggest that the DLPFC–caudate pathway can be a neuroimaging marker implicated in the potential mechanism for noninvasive brain stimulation for smoking [52].

## 4 Limitations

This study had several limitations. First, rTMS decreased the craving for cigarette smoking for a short time; however, after rTMS was stopped, the cravings recovered in 2 weeks. Second, because it is difficult to find female patients with TUD in China, we only included male patients with TUD in our study. Third, the sample demographics of the current study (young, male, low nicotine dependence) might limit the generalizability of our findings. Fourth, whether the effect of smoking cues could effectively elicit smoking craving for TUD needs testing before the experiment. Another limitation concerns the absence of an additive behavioral effect of pre‐stimulation craving priming. This may reflect that the brief 5‐min video exposure was insufficient to induce a stable motivational state capable of interacting synergistically with rTMS, especially given the rapid decay of cue‐induced craving and the cognitive demands of the subsequent Go/No‐go task. In addition, substantial individual variability in cue reactivity and the possibility that longer or repeated priming is required for state‐dependent neuromodulation effects may have diluted group‐level behavioral gains.

## 5 Conclusions and Clinical Implications

The current study confirmed the effectiveness of rTMS in reducing craving for smoking and further revealed how rTMS enhanced top‐down control by reshaping the prefrontal–striatal pathway. This provides a theoretical base for the application of rTMS in the treatment of craving for smoking. This study is valuable in understanding the neural mechanism regarding how rTMS functions in TUD, and it provides insights into finding effective treatment strategies for TUD.

## Author Contributions

Xin Luo, Shuang Li and Hongan Chen analysed the behavioural, task‐fMRI and correlation data and prepared the relevant figures; Xuefeng Ma analysed the functional connectivity data and prepared the relevant figures; Yanbin Zheng and Bo Yang contributed to data collection. Guang‐Heng Dong designed the entire study and revised the manuscript.

## Funding

The current research was supported by the Yunnan Provincial Department of Science and Technology Basic Research Project (202405AC350075; 202501AS070042) and the Innovation Team Program in Philosophy and Social Science of Yunnan Province (Research on psychological adaptation and development of China‘s ethnic minority students in border areas). The funding agencies did not contribute to the experimental design or conclusions, and the views presented in the manuscript are those of the authors and may not reflect the funding agencies.

## Conflicts of Interest

The authors declare no conflicts exist.

## Supporting information


**Table S1:** Correlation of FC changes (post‐pre) with diagnostic changes in FTND and TQSU.
**Table S2:** Correlation of FC changes (post‐pre) with diagnostic changes in FTND and TQSU.

## Data Availability

The data that support the findings of this study are available from the corresponding author upon reasonable request.
