# Supplementary material for: rTMS Suppresses Tobacco Craving via Enhanced Prefronto‐Striato‐Thalamic Connectivity
Source: Addict Biol. 2026 May 14;31(5):e70167. doi: 10.1111/adb.70167 (PMC13172771; doi:10.1111/adb.70167)
Supplement: Supplementary file 1 — Table S1: Correlation of FC changes (post‐pre) with diagnostic changes in FTND and TQSU. Table S2: Correlation of FC changes (post‐pre) with diagnostic changes in FTND and TQSU. [file ADB-31-e70167-s001.docx]

**Supplementary materials for:**

**rTMS Suppresses Tobacco Craving via Enhanced Prefronto-Striato-Thalamic Connectivity**

**Behavioral measures**

In the current study, we did not ask subjects to response as soon as possible (response in 2 seconds). On RT, we did not observe significant group (active, sham) * tests (pre-, post-) effect (*F*=2.297, *p*>0.05, ƞ^2^=0.131). In accuracy rates, both groups showed higher than 90% and also no group difference was observed (*F*=1.336, *p*>0.05, ƞ^2^=0.142). In the current study, we only included the accurate response for fMRI data analyses.

**Post-hoc analysis on FTND (Continued with the main finding)**

No group difference was observed in pre-test (*p*=0.282, ƞ^2^=0.901); Significant group difference was observed in post-test (*p*=0.030, ƞ^2^=0.213).

**The TQSU scores**

Further analyses revealed that in the rTMS group, the rTMS decreased the TQSU score significantly in post-tests (pre [M=26.38, SD=1.241**]** and post [M=13.41, SD = 0.821**];** *p*<0.001, ƞ^2^=0.233). This effect was not observed in the sham group (pre [M=26.682, SD=1.331**]** and post [M=23.976, SD=1.407**];** *p*=0.251, ƞ^2^=0.111). No group difference was observed in pre-test (*p*=0.371, ƞ^2^=0.894); Significant group difference was observed in post-test (*p*<0.0001, ƞ^2^=0.225).

**Supplementary table 1. Correlation of FC changes (post-pre) with diagnostic changes in FTND and TQSU**

|  | | L_ACC-  L_Thalamus | L_Caudate-  L_Thalamus | L_Caudate-  L_DLPFC | L_Thalamus-  R_Caudate |
| --- | --- | --- | --- | --- | --- |
| FTND | *r* | 1.000** | 0.122 | -0.192 | -0.296 |
|  | *p* | 0 | 0.506 | 0.292 | 0.1 |
| TQSU | *r* | -0.26 | .614** | -.757** | 0.061 |
|  | *p* | 0.151 | 0 | 0 | 0.739 |

DLPFC = Dorsolateral Prefrontal Cortex; ACC = Anterior Cingulate Cortex;

FTND = Fagerstrom Test of Nicotine Dependence; TQSU = Tiffany Questionnaire for Smoking Urges.

**. *p*<0.01; *. *p*<0.05.

**Supplementary table 2. Correlation of FC changes (post-pre) with diagnostic changes in FTND and TQSU**

|  | | L_DLPFC-  L_precuneus | L_DLPFC-  L_basal_ganglia | L_DLPFC-  R_basal_ganglia | L_DLPFC-  L_thalamus | L_DLPFC-  R_parietal |
| --- | --- | --- | --- | --- | --- | --- |
| FTND | *r* | 0.108 | -0.04 | 0.188 | -.406* | -0.328 |
|  | *p* | 0.557 | 0.83 | 0.303 | 0.021 | 0.066 |
| TQSU | *r* | -0.03 | -.390* | 0.043 | 0.265 | 0.258 |
|  | *p* | 0.872 | 0.027 | 0.814 | 0.142 | 0.154 |

DLPFC = Dorsolateral Prefrontal Cortex; FTND = Fagerstrom Test of Nicotine Dependence; TQSU = Tiffany Questionnaire for Smoking Urges.

**. *p*<0.01; *. *p*<0.05.
